# 伴腋窝淋巴结寡转移的晚期胸腺肠型腺癌1例

**DOI:** 10.3779/j.issn.1009-3419.2026.101.18

**Published:** 2026-08-20

**Authors:** Chuan HUANG, Zheng WANG, Hongfeng TONG

**Affiliations:** 1 100730 北京，北京医院（国家老年医学中心）胸外科，老年疾病国家临床医学研究中心，国家卫生健康委老年医学重点实验室，中国医学科学院老年医学研究院; 1 Department of Thoracic Surgery; 2 病理科; 2 Department of Pathology, Beijing Hospital, National Center of Gerontology;National Clinical Research Center for Gerontology; The Key Laboratory of Geriatrics of NHC; Institute of Geriatric Medicine,Chinese Academy of Medical Sciences, Beijing 100730, China

**Keywords:** 胸腺肿瘤, 肠型腺癌, 寡转移, 外科手术, 综合治疗, Thymic neoplasms, Enteric-type adenocarcinoma, Oligometastasis, Surgical procedures, Multimodality therapy

## Abstract

胸腺肠型腺癌（enteric-type thymic adenocarcinoma, ETAC）是胸腺癌的一种罕见亚型，其组织学和免疫表型与肠道腺癌高度相似，易误诊为消化道转移癌。本文报道1例伴腋窝淋巴结寡转移的晚期ETAC患者，经正电子发射断层显像/计算机断层扫描（positron emission tomography/computed tomography, PET/CT）及胃肠镜排除消化道原发肿瘤，患者接受正中开胸胸腺扩大切除术，有效缓解上腔静脉梗阻与心肺压迫症状，术后行全身化疗联合免疫治疗，以及针对原发瘤床和转移淋巴结的区域放疗。术后随访24个月未见疾病进展。本例表明对于伴有寡转移的晚期ETAC患者，积极的局部治疗联合基于分子分型的全身治疗有可能为部分经过严格选择的寡转移患者提供新的治疗机会。

## Abstract (en)

Enteric-type thymic adenocarcinoma (ETAC) is a rare subtype of thymic carcinoma. It shares highly similar histological and immunophenotypic features with intestinal adenocarcinoma, and is therefore prone to being misdiagnosed as metastatic carcinoma of gastrointestinal origin. Herein, we report a case of advanced ETAC with axillary lymph node oligometastasis. Positron emission tomography/computed tomography (PET/CT) and gastrointestinal endoscopy were performed to rule out primary gastrointestinal malignancy. The patient underwent median sternotomy with extended thymectomy, which effectively relieved superior vena cava obstruction and cardiopulmonary compressive symptoms. Postoperatively, systemic chemotherapy combined with immunotherapy, as well as regional radiotherapy targeting the primary tumor bed and metastatic lymph nodes, were administered. The patient remained disease-free during the 24-month postoperative follow-up. This case suggests that aggressive local therapy combined with molecular-subtype-based systemic treatment may offer novel therapeutic opportunities for carefully selected patients with advanced ETAC and oligometastases.

胸腺肠型腺癌（enteric-type thymic adenocarcinoma, ETAC）是胸腺癌中一种罕见亚型，其组织学形态及免疫表型与肠道腺癌高度相似。2015年Moser等^[1]^首次提出ETAC是胸腺癌的一种独立亚型，2021年世界卫生组织（World Health Organization, WHO）将其定义为兼具结直肠腺癌样组织学形态（腺管状/筛状结构）与肠源性免疫表型的原发性胸腺恶性肿瘤^[2]^。截至2020年全球仅报道33例符合WHO标准的ETAC^[3]^，关于其生物学行为、手术指征、最佳治疗模式及预后特征尚不明确。本文旨在通过1例伴有远处寡转移、经多学科综合治疗后获得长期生存的ETAC病例，结合文献探讨ETAC的临床病理特征、手术方案及个体化诊疗策略。

## 1 病例资料

### 1.1 一般资料与临床表现

患者男，62岁，于2024年5月27日就诊于北京医院，主诉为“反复胸前区疼痛伴咳嗽2年，加重伴发作性晕厥3天”。患者2年前出现劳累后胸前区阵发性压榨样疼痛，放射至左肩背部，伴偶干咳，休息后可缓解，患者未进一步诊治。3天前患者剧烈咳嗽后突发晕厥，共发作3次，每次持续3-5 min后自行苏醒，伴头晕、乏力，无四肢抽搐、大小便失禁等。胸部计算机断层扫描（computed tomography, CT）示前纵隔不规则肿物，外侵明显。心脏超声示少-中量心包积液，心包穿刺引流出血性积液，细胞病理未见癌细胞。本研究获得北京医院伦理委员会批准（No.2026BJYYEC-KY133-01）。

### 1.2 辅助检查

#### 1.2.1 肿瘤分期检查

2024年5月29日胸部增强CT（图1）示前上纵隔不规则肿物，密度不均匀，增强扫描呈中度强化；肿物包绕升主动脉及左侧无名静脉，致左侧无名静脉管腔狭窄甚至闭塞，侧支循环建立；肿物与心包分界不清，伴心包不均匀增厚及双侧少量胸腔积液。2024年5月30日正电子发射断层显像/CT（positron emission tomography/CT, PET/CT）示前纵隔肿物代谢显著增高，最大标准化摄取值（maximum standardized uptake value, SUVmax）为18.5；左侧腋窝肿大淋巴结，短径1.2 cm，放射性摄取异常增高（SUVmax为20.7），考虑转移；余全身未见其他高代谢病灶。胃镜示反流性食管炎、慢性非萎缩性胃炎，结肠镜示结肠多发息肉，病理为管状腺瘤及增生性息肉。血清肿瘤标志物：糖类抗原（carbohydrate antigen, CA）125为366.8 U/mL，CA72-4为27.4 U/mL，细胞角蛋白19片段为18.9 ng/mL，神经元特异性烯醇化酶为21.1 ng/mL，癌胚抗原、甲胎蛋白及CA19-9在正常范围。左侧腋窝淋巴结行超声引导下穿刺活检病理为低分化腺癌浸润，免疫组化：细胞角蛋白 20（cytokeratin 20, CK20）（+）、尾型同源盒转录因子 2（caudal-type homeobox transcription factor 2, CDX2）（+）、甲状腺转录因子1（thyroid transcription factor-1, TTF-1）（-）、CK7（+），结合前纵隔肿物及排除胃肠道原发灶后，倾向ETAC转移。Masaoka-Koga分期为IVB期，肿瘤原发灶-淋巴结-转移（tumor-node-metastasis, TNM）（参照第九版胸腺肿瘤TNM分期标准）分期为cT3N0M1b，IVB期。本病例通过PET/CT和胃肠镜检查排除胃肠道原发肿瘤，符合原发性ETAC的诊断标准。

**图1:**
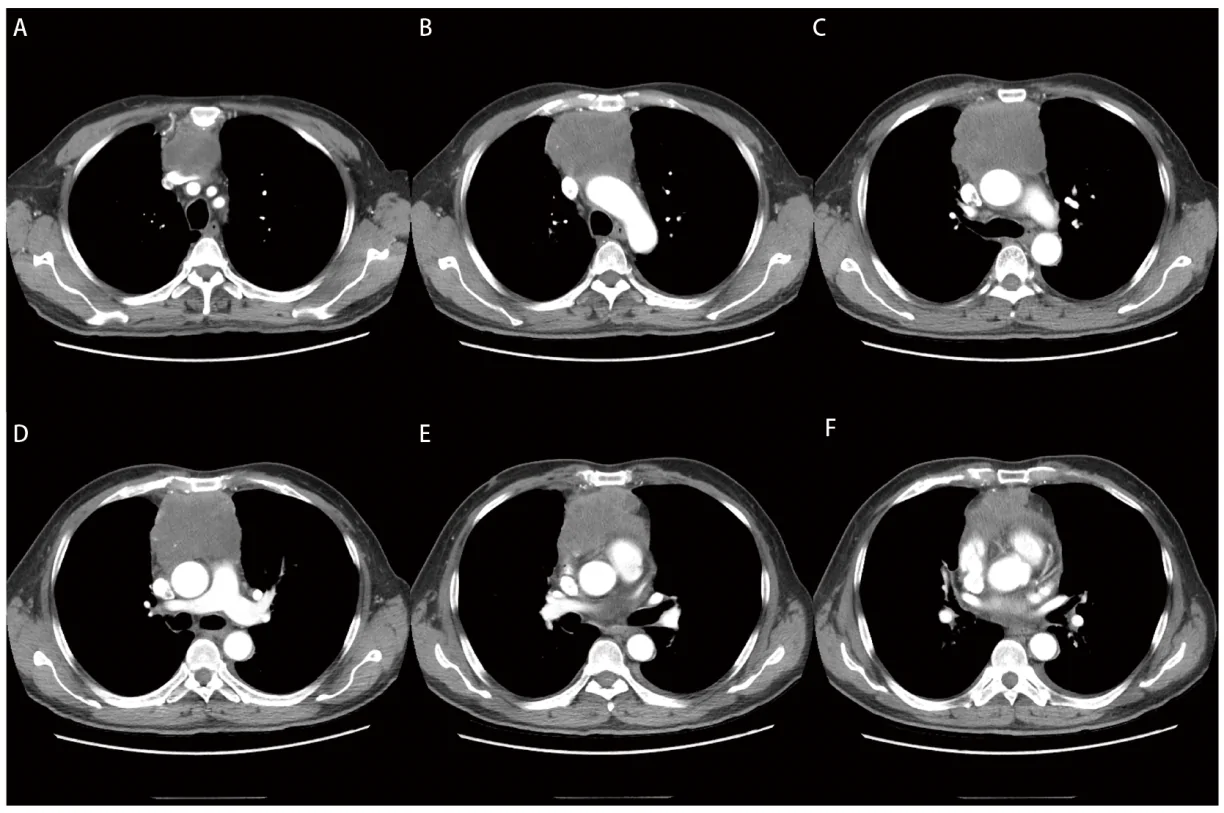
胸部增强CT显示前上纵隔肿物，大小约8.7 cm×5.4 cm，边缘呈分叶状，密度不均匀，增强扫描呈中度强化；肿物包绕升主动脉及左侧无名静脉，致左侧无名静脉管腔狭窄甚至闭塞，侧支循环建立（A-D）；肿物与心包分界不清，心包不均匀增厚，双侧少量胸腔积液（E-F）。

#### 1.2.2 心肺功能及其他检查

超声心动：左室射血分数56%，少-中量心包积液，液性暗区7-9 mm，主动脉瓣钙化（轻度），二尖瓣、三尖瓣关闭不全（轻度）。肺功能：重度混合性通气功能障碍，以阻塞性为主，小气道功能障碍，弥散功能障碍；用力肺活量（forced vital capacity, FVC）为2.23 L（占预计值59.8%）；第1秒用力呼气容积（forced expiratory volume in one second, FEV_1_）为1.08 L（占预计值36.9%）；FEV_1_/FVC为48.64%；最大通气量（maximal voluntary ventilation, MVV）为43.56 L/min（占预计值38.9%）；一氧化碳弥散量为5.59 mmol/（min·kPa）（占预计值65.4%）。重复神经电刺激及单纤维肌电图结果为阴性，结合神经专科查体，排除重症肌无力。

### 1.3 手术及术后病理

#### 1.3.1 手术经过

2024年6月4日，患者在全麻下行正中开胸入路胸腺肿瘤及胸腺扩大切除、区域淋巴结清扫术，术中见肿瘤上方侵犯左侧无名静脉下壁致管腔狭窄，切除受累无名静脉壁并取自体心包补片修补；肿瘤与主动脉外膜粘连但未直接侵犯；肿瘤两侧侵犯左肺上叶及右肺中叶，楔形切除部分受侵肺组织，胸膜腔内未见肿瘤种植转移；肿瘤后方侵犯心包，心包内少量血性积液和纤维粘连，心包内未见肿瘤种植转移，切除受累心包、松解心包内粘连；颈根部至胸腺上极、上腔静脉后壁至气管旁、无名静脉旁、膈神经旁、主肺动脉窗等区域探及多枚淋巴结，清扫上述区域淋巴结。左腋窝转移淋巴结未手术切除，后续接受根治剂量放疗。手术历时155 min，术中出血约200 mL，输注红细胞2 U。

#### 1.3.2 术后病理

ETAC，低分化，肿瘤最大径12.0 cm，侵犯胸腺脂肪、胸膜、肺组织及无名静脉壁，肺组织切缘、心包切缘未见癌累及，原发灶手术切缘阴性；纵隔淋巴结未见转移癌（0/18，其中颈根部淋巴结0/1，右侧喉返神经旁淋巴结0/5，左侧膈神经旁淋巴结0/4，无名静脉旁淋巴结0/2，上腔静脉后淋巴结0/2，主动脉旁淋巴结0/3，左侧心隔角淋巴结0/1）。病理分期：pT3N0M1b，IVB期，Masaoka-Koga IVB期。免疫组化：CD5（-），CD117（-），AE1/AE3（+++），CK7（+++），CK20（++），CK5/6（-），P40（-），CDX2（+++），特殊AT富集序列结合蛋白2（special AT-rich sequence-binding protein 2, SATB2）（+），CD56（-），Syn（-），ChgA（-），TTF-1（-），Sall4（-），OCT3/4（-），P53（符合突变型），Ki67（80%+），TDT（-）。部分免疫染色见图2。分子病理：下一代测序显示肿瘤突变负荷（tumor mutational burden, TMB）为8.64 Muts/Mb，微卫星稳定，检出*TP53*（p.G245D）、*CDK12*（p.R784*）及*BRCA1*缺失等体细胞变异，程序性死亡配体 1（programmed death-ligand 1, PD-L1）检测（检测试剂克隆号Roche/VENTANA SP263）示肿瘤细胞<1%，免疫细胞（IC+）10%。

**图2:**
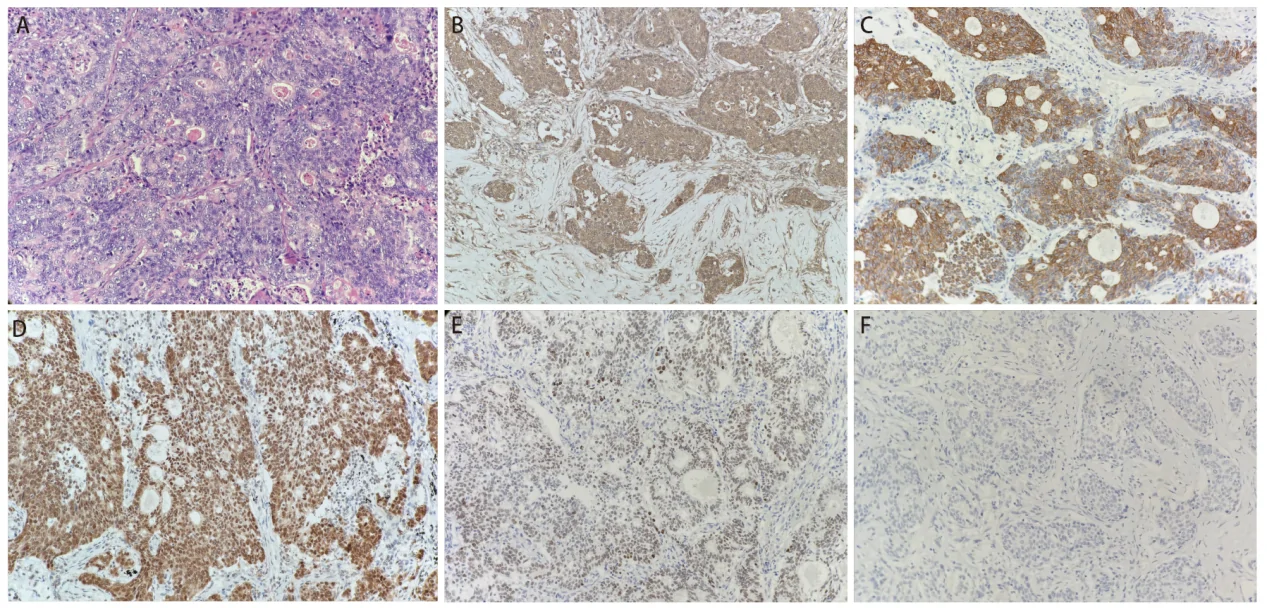
肿瘤排列呈实性巢团、筛状及腺管状，可见腺腔内坏死，肿瘤细胞呈高柱状，核分裂象多见，异型性明显，形态类似于结直肠癌，低分化，肿瘤侵犯胸腺脂肪组织，并累及邻近肺实质。肿瘤细胞呈肠源性分化，包括CK20、CDX2及SATB2，尽管CD5及CD117染色阴性，结合临床符合胸腺来源肠型腺癌（×200）。A：肿物镜下排列为筛状、腺样，筛孔中央可见坏死, 细胞呈高柱状，异型性明显，核分裂象多见，形态与结直肠癌相似（HE染色）；B：CK7（+++）（IHC染色）；C：CK20（+++）（IHC染色）；D：CDX2（+++）（IHC染色）；E：SATB2（+）（IHC染色）；F：TTF-1（-）（IHC染色）。

### 1.4 术后治疗与随访

患者术后无肺部感染、心律失常、神经损伤、乳糜胸等并发症，术后住院时间12天。待手术康复后于术后第60天开始行化疗联合免疫治疗共4个周期，方案为白蛋白结合型紫杉醇100 mg/m²（d1, d8, d15）+卡铂曲线下面积（area under the curve, AUC）=5（d1）+信迪利单抗200 mg（d1），每21天为1个周期。化免治疗结束后于术后第141天开始行辅助放疗，靶区包括瘤床[临床靶体积（clinical target volume, CTV）1]、纵隔及左腋窝淋巴结引流区（CTV2/CTVn），剂量为：CTV1（瘤床）60 Gy/28次；CTV2（3A淋巴结引流区）50.4 Gy/28次；肿瘤靶体积（gross tumor volume, GTV）n（左腋窝转移淋巴结）60 Gy/28次；CTVn（左腋窝周围引流区）50.4 Gy/28次。患者术后一般状况良好，随访24个月未见疾病进展，胸腔手术区域及全身脏器未见新发转移灶，CT影像显示左侧腋窝淋巴结较前明显缩小（淋巴结短径：手术前为12 mm，化免治疗4个周期后为5 mm，放疗结束后为5 mm，术后24个月为5 mm）。

## 2 讨论

### 2.1 ETAC的临床病理特征

ETAC是一种具有肠道分化特征的胸腺癌，其发病机制尚不明确，可能与胸腺内残留的胚胎性多能干细胞向肠上皮分化有关。ETAC好发于中老年男性，男女比例约2:1，常缺乏特异性症状，肿瘤压迫可导致咳嗽、胸痛、呼吸困难、上腔静脉阻塞综合征等^[1,4]^。本例患者起病症状为胸痛、咳嗽后晕厥，系肿瘤压迫上腔静脉系统及心包积液所致，肿瘤侵袭性强，病情凶险。

ETAC在病理上多表现为黏液性腺癌，常表达CK20和/或CDX2，多数病例TTF-1阴性，CD5和CK7的阳性率约为50%^[3]^。确诊原发性ETAC须满足以下条件：（1）组织学及免疫表型符合肠型腺癌特征；（2）通过胃肠镜、PET/CT等排除胃肠道及其他部位（如肺、胰胆管）的原发灶；（3）符合WHO胸部肿瘤分类中关于胸腺癌的诊断标准。本例通过PET/CT、胃肠镜等辅助检查排除消化道及其他隐匿病灶。此外，ETAC还需与胸腺癌其他少见亚型如黏液表皮样癌、NUT癌、胸腺神经内分泌肿瘤等鉴别，本例P40（-）及典型的肠型免疫表型支持ETAC的诊断。

ETAC预后较差，其生存期显著短于其他类型胸腺癌。Conforti等^[3]^总结33例ETAC为目前最大宗报道，按Masaoka分期标准包括11例I-II期、10例III期、12例IV期，其中局限期ETAC中位无进展生存期为12个月，IV期患者无进展生存期仅3-5个月，全组患者中位总生存期仅34个月。Jung等^[5]^报道ETAC的平均生存期[（85.6±23.1）个月]显著优于非肠型腺癌[（22.1±7.4）个月]，Kwon等^[6]^认为黏液性及非特指型亚型胸腺腺癌预后较差，Hamanaka等^[2]^报道黏液性腺癌与非黏液性腺癌的无病生存期与总生存期无显著差异，结论互不一致。涉及ETAC的多数研究均为个案报道或小样本研究，不同研究在以下方面存在显著异质性：（1）纳入标准不统一，部分研究将混合性腺癌或伴肠型分化的胸腺癌一并纳入；（2）病例分期差异大，涵盖I-IVB期；（3）手术切除完整性（R0/R1/R2）及全身治疗方案（化疗方案、是否联合放疗/免疫治疗）各不相同。因此，不同文献报道的生存数据不宜直接横向比较。

### 2.2 ETAC的综合治疗策略

ETAC侵袭性高，易侵犯压迫胸腔内重要脏器，还易转移至肝、肺、肾、肾上腺、脑等多个部位^[2]^。手术完整切除是胸腺癌治疗的基石，但对于已经出现远处转移（如淋巴结、远隔脏器）的IV期患者，无法达到完整切除，远期预后差。本例患者远处转移灶仅限于单一区域淋巴结（左侧腋窝），纵隔淋巴结及全身其他脏器无转移，符合寡转移概念，经多学科讨论认为积极局部治疗联合全身治疗有望给患者带来临床获益，并且因原发肿瘤体积大、对周围组织外侵压迫明显，已导致较严重的上腔静脉梗阻、心包积液、呼吸功能受损，患者生活质量极差，已出现多次晕厥病史，随时可能发生猝死，极易再出现心包填塞、意识丧失等致命性并发症。因此决定手术切除原发肿瘤及纵隔淋巴结，旨在解除压迫症状，降低肿瘤负荷，为后续全身综合治疗创造条件。

值得重视的是，此类患者呼吸、循环功能已严重减损，围术期各类心肺并发症风险极高，术前应组织多学科会诊对全身脏器功能进行充分且谨慎的评估，术中和术后应联合麻醉科、重症医学科等进行严密管理，有效预防围术期并发症。本例纵隔淋巴结无转移，腋窝淋巴结转移呈“跳跃性”分布，其可能机制包括：肿瘤累及左侧无名静脉致回流受阻，经胸壁侧支循环及肋间淋巴通路上行转移；纵隔淋巴引流与腋窝淋巴链存在潜在交通。该转移模式在胸腺癌中较罕见，提示ETAC具有独特生物学行为。

ETAC病例全球罕见，目前尚无标准化疗方案，既往报道多参考胸腺癌（如CAP方案：顺铂+阿霉素+环磷酰胺）或结直肠癌方案（如FOLFOX方案：氟尿嘧啶+亚叶酸钙+奥沙利铂），但传统方案有效率较低，IV期患者中位无进展生存期仅3-5个月^[3]^。本例术后采取全身治疗药物联合局部放疗的治疗策略。因心肺功能储备差，化疗方案弃用含蒽环类药物的CAP方案，选择了心脏毒性相对可控且对腺癌有效的紫杉醇联合卡铂方案。Conforti等^[3]^研究未评估TMB或PD-L1等免疫治疗生物标志物及免疫治疗在ETAC全身治疗中的疗效。已有II期临床研究^[7,8]^提示铂类为基础的化疗联合程序性死亡受体1（programmed death-1, PD-1）/PD-L1抑制剂治疗III-IV期胸腺癌的客观缓解率可达55%-56%，展现出良好的疾病控制潜力。因此本例在标准化疗方案基础上联合PD-1单抗，目前无进展生存期达24个月，提示基于分子分型的免疫治疗有望为此类难治性肿瘤开辟新的治疗路径。

本例放疗靶区覆盖了原发瘤床（60 Gy）、纵隔淋巴引流区及腋窝淋巴结（50.4-60 Gy），尽管腋窝淋巴结被视为M1病变，但在原发灶切除且全身治疗有效的前提下，对寡转移灶进行根治量放疗仍有望提高局部控制率。本例随访至术后24个月仍保持无瘤生存，提示“手术减瘤+区域放疗+全身药物”的综合治疗模式在经严格筛选的晚期ETAC病例具有可行性与潜在优势，未来仍需更多病例和长期随访验证。

综上所述，ETAC是一种易误诊为消化道转移癌的罕见胸腺恶性肿瘤，对于伴有寡转移的晚期ETAC患者，尽管总体预后不佳，但在多学科团队严格评估下，针对原发灶的积极减瘤手术，联合基于分子病理特征指导的全身药物治疗，以及对寡转移灶的精准放疗，有可能为部分经过严格选择的寡转移患者提供新的治疗机会。
